# Influence of peripartum on the erythrogram of Holstein dairy cows

**DOI:** 10.4102/jsava.v91i0.1975

**Published:** 2020-06-17

**Authors:** Renan B. Paiano, Daniela B. Birgel, Eduardo H. Birgel Junior

**Affiliations:** 1 Department of Animal Reproduction, School of Veterinary Medicine and Animal Sciences, University of São Paulo, São Paulo, Brazil; 2 Department of Veterinary Medicine, Faculty of Animal Science and Food Engineering, University of São Paulo, São Paulo, Brazil

**Keywords:** haematology, body condition score, milk production, lactating Holstein dairy cows, veterinary

## Abstract

**Background:**

Peripartum is a challenging phase for the health of cows.

**Objectives:**

This study analysed the haematological profile of Holstein cows during peripartum.

**Method:**

Blood samples were collected on days 18, 12, 8, 5 and 2 before calving, at parturition, and on days 1, 7, 14, 21, 30, 45 and 60 postpartum. Analyses of red blood cell (RBC) count, haemoglobin concentration, haematocrit, mean corpuscular volume, mean corpuscular haemoglobin, mean corpuscular haemoglobin concentration and RBC distribution width were performed; body condition score (BCS) and milk yield were evaluated.

**Results:**

Red blood cell (the highest value was 6.10 × 10^12^/L at parturition and the lowest recorded value was 5.03 × 10^12^/L 21 days after parturition), haemoglobin and haematocrit (the highest values were 10.48 g/dL and 33.47% at parturition, respectively and the lowest values were 8.28 g/dL and 26.13% on day 30 after parturition, respectively); BCS (the highest and the lowest values were 3.50 points and 2.73 points on days 18 before parturition and 45 after parturition, respectively) and milk production (the lowest and the highest values were 21.48 L and 27.02 L on days 7 and 45 after parturition, respectively) were significantly different (*p* < 0.05) during the peripartum period. Of the total cows (*n* = 48), 41.7% had RBC, haemoglobin and haematocrit below the reference intervals during at least one collection point during the postpartum period.

**Conclusion:**

This study demonstrated that dairy cows included in this investigation suffered alterations in select haematological variables during the postpartum period.

## Introduction

The peripartum period comprises the last 3 weeks of gestation to 3 weeks postpartum (Grummer 1995). During this period, homeorrhetic adaptations occur in preparation for parturition and the onset of lactation (Bauman & Currie 1980). These endocrine, metabolic and immunological changes include increased circulating concentrations of oestrogen, non-esterified fatty acids (NEFA) and beta-hydroxybutyrate (BHB), and reduced serum concentrations of progesterone, calcium, glucose, insulin and insulin-like growth factor 1 (IGF-1) (Drackley 1999; Kimura et al. 2002; Meglia et al. 2005; Moreira et al. 2015; Paiano et al. 2018, 2019a, 2019b, 2019c, 2019d; Wankhade et al. 2017). The growth of the foetus in late gestation can cause compression and increased internal pressure of digestive organs because of decreased physical space, resulting in the reduction of dry matter intake and the negative energy balance (Bell 1995; Goff & Horst 1997; Ingvartsen & Andersen 2000; Kaufman et al. 2017). During this phase, there is an increase in the demand for nutrients to support colostrogenesis, lactogenesis and foetal growth, intensifying the negative energy balance, and it becomes necessary to mobilise body fat to meet this deficit (Bell 1995; Drackley 1999). As a result, there is an increased mobilisation of NEFA, as well as increased uptake by the liver, leading to a greater accumulation of triglycerides in the hepatic cytosol. This can lead to fatty liver, which increases the risk of developing various diseases in the postpartum period (Caixeta et al. 2017; Herdt 2000; Jeong et al. 2018; Paiano et al. 2020; Sheehy et al. 2017).

Owing to the above-mentioned adaptations, the peripartum period is a challenging phase for the health of a dairy cow. It is the most critical period of a dairy cow’s production cycle, as it determines the success or failure of the future productive and reproductive performance. In view of the changes that occur during the peripartum and the increase in the incidence of diseases during this phase, it is essential to monitor the health of the herd by various means, including through blood tests, and to carry out any interventions if necessary. In this sense, evaluation of erythrogram is useful for assessing animal health, especially in terms of detecting the presence of anaemia of various aetiologies. Drastic physiological changes, such as those occurring in the peripartum period, may significantly influence various haematological measures. Thus, in order to avoid erroneous diagnoses, knowledge of the effect of any physiological changes on the erythrogram could be useful when interpreting results. Moreover, with the exception of haematocrit, the haematological profile of Holstein dairy cows during peripartum has not been described previously in detail. Thus, this study aimed to characterise the haematological profile of Holstein cows during peripartum.

## Materials and methods

At the beginning of this study, all cows were examined clinically, and only healthy cows were used. Forty-eight Holstein multiparous dairy cows (parity: 2.9 ± 0.4) were included in this study. The cows were housed in a freestall barn, fed a total mixed feed system (total mixed ration [TMR]) twice a day and milked twice daily. The prepartum diet comprised 17 kg of corn silage and the concentrate comprised 2.72 kg of ground corn, 800 g of soybean meal and 400 g of anionic salt (Minerthal nucleus milk prepartum^®^, Minerthal, Brazil). The nutritional content was 42.36% of neutral detergent fibre, 14.71% crude protein and 3.03% of fat. The postpartum diet consisted of 27.5 kg of corn silage, 5.18 kg of corn ground, 2.890 kg of soybean meal and 180 g of postpartum mineral supplement (Minerthal nucleus milk MD^®^). The nutritional content was 41.78% of neutral detergent fibre, 16.5% crude protein and 4% of fat.

Blood samples were taken at 0700 hours before feeding on days 18, 12, 8, 5 and 2 before the predicted calving date, at parturition (3 hours – 8 hours after parturition), and on days 1, 7, 14, 21, 30, 45 and 60 postpartum. Blood was collected by coccygeal venipuncture and placed into a vacuum glass tube containing ethylenediaminetetraacetic acid (EDTA) as an anticoagulant (Vacutainer Systems, Becton Dickinson, Franklin Lakes, NJ). Samples were analysed at the Multiuser Laboratory in Clinical Veterinary Analysis of the University of São Paulo. Blood samples were transported to the laboratory and analysed within 3 h of collection. Analyses of red blood cell count (RBC), haemoglobin concentration (Hb), haematocrit (Ht), mean corpuscular volume (MCV), mean corpuscular haemoglobin (MCH), MCH concentration (MCHC) and RBC distribution width (RDW) were performed using a veterinary haematology analyser (BC-2800 Vet Mindray) with species-specific settings for cattle.

Cows had their body condition score (BCS) assessed at all time points by a single person using a 1–5 scale (Ferguson, Galligan & Thomsen 1994). Milk yield was recorded at each milking and saved in the software program Alpro (DeLaval, Tumba, Sweden).

Statistical analysis was performed using statistical analysis software (SAS) (version 9.3 SAS/STAT; SAS Institute Inc., Cary, NC). After testing for normality with the Shapiro–Wilk test, an analysis of variance (ANOVA) was performed for erythrocyte indices, milk production and BCS, using the mixed-model procedure for repeated measurements (PROC MIXED) of SAS. The model included variable days as a fixed effect and the variable parity and cow as a random effect. Mean values were compared with Tukey’s test. Differences with *p* < 0.05 were considered significant. Values are presented as the mean ± standard error (SE).

### Ethical considerations

The study was conducted on the farm of the University of São Paulo, Fernando Costa Campus, located in the city of Pirassununga, Brazil. All animal procedures were approved by the Animal Use Ethics Committee of the Faculty of Veterinary Medicine and Animal Science of the University of São Paulo (protocol number: 8022150216).

## Results

**FIGURE 1:**
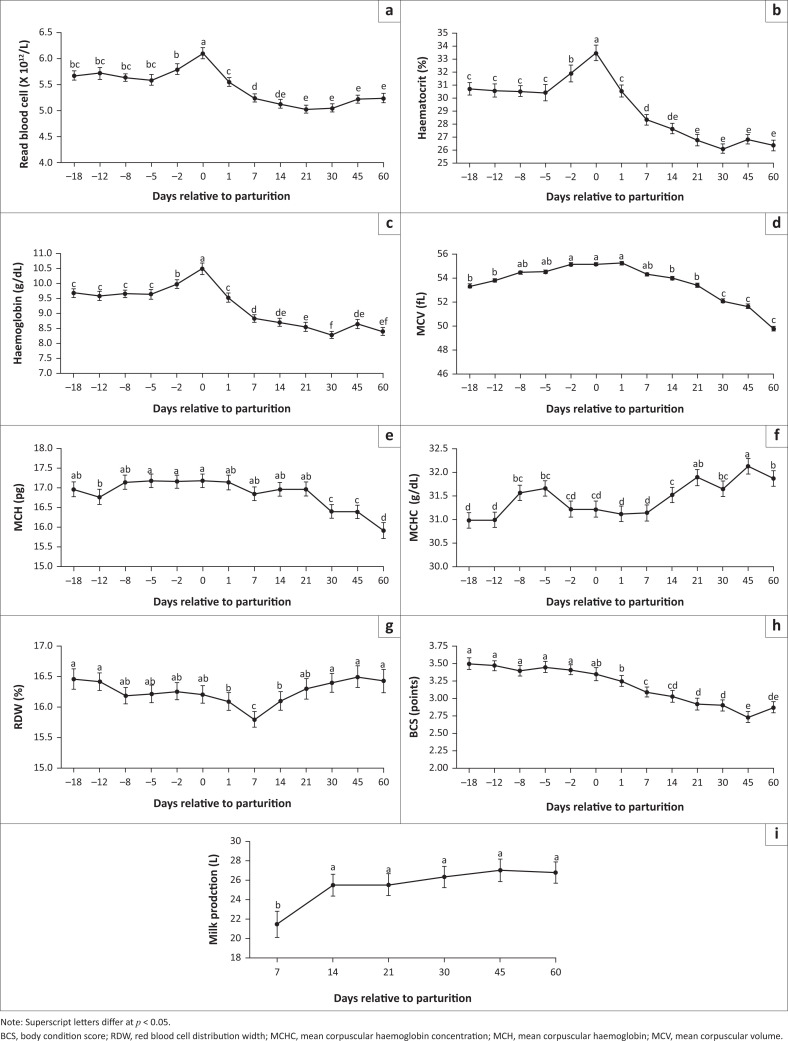
Mean (±SE) (a) red blood cell (×10^12^/L), (b) haematocrit (%), (c) haemoglobin (g/dL), (d) mean corpuscular volume (fL), (e) mean corpuscular haemoglobin (pg), (f) mean corpuscular haemoglobin concentration (g/dL), (g) red blood cell distribution width (%), (h) body condition score (points) and (i) milk production (L) of Holstein dairy cows during peripartum. Superscript letters differ at *p* < 0.05.

Figure 1 shows the results of the haematological profile, body condition score and milk production of dairy cows during the peripartum period. Red blood cells, Hb and Ht were significantly different (*p* < 0.05) at different time points during the peripartum period. The lowest value of RBC was observed on 21 days postpartum (5.03 × 10^12^/L), which differed significantly (*p* < 0.05) from the values observed in the prepartum period. For Ht and Hb, the lowest values were observed at 30 days postpartum, corresponding to 26.13% and 8.28 g/dL, respectively; these values differed from those observed during the prepartum period. The lowest values of erythrocyte indices, observed at 60 days after parturition, differed (*p* < 0.05) from the values observed in the prepartum period, with averages of 49.76 femtolitres (fL) and 15.91 picograms/cell (pg) for MCV and MCH, respectively, whereas the lowest value of MCHC was noted at 18 days before parturition (30.98 g/dL). The lowest values of RDW were observed at 7 days postpartum (15.8%), differing (*p* < 0.05) from the values at other periods.

The mean BCS values of animals between 1 and 60 days after parturition were significantly lower (*p* < 0.05) than the values from the prepartum period. The highest value of BCS was noted at 18 days before parturition (3.50 points) and the lowest value was observed at 45 days after parturition (2.73 points). Regarding milk production, only the production on day 7 (21.48 L) was lower (*p* < 0.05) than other mean production values during the postpartum period.

## Discussion

The increased RBC count, increased concentration of Hb and increased Ht at parturition are indicative of hemoconcentration that probably occurred because of increased physical effort during parturition and a decrease in water intake. Of the total cows, 41.7% (20/48) showed RBC, Hb and Ht below the reference intervals (RBC, Hb and Ht lower than 5.0 × 10^12^/L, 8.0 g/dL and 24%, respectively) (Radostits et al. 2007) during at least one collection time point during the postpartum period. No animals had jaundice or fever, and the blood smear evaluations ruled out the possibility that the lower RBC, Ht and Hb values were caused by haemolytic parasites (*Anaplasma marginale* or *Babesia* spp.). Anaemia was unlikely to be because of gastrointestinal parasites as the animals were dewormed at the beginning of the study. During postpartum, there was a gradual reduction in the values of MCV and MCH; however, the values observed at 60 days postpartum were still within the reference intervals and characterised as normocytic normochromic.

Reduction in Ht, RBC and Hb values and the occurrence of anaemia during the puerperal period have not been extensively documented previously for dairy cows. Saut and Birgel Junior (2008) observed lower erythrogram values for cows with retained placenta during postpartum period when compared with healthy cows. Amongst the possible causes of postpartum anaemia, we assumed that this finding could be associated with an increased production of pro-inflammatory cytokines which modulated inflammatory response (Chikazawa & Dunning 2016). Cytokines could cause reduced erythropoiesis, most likely through inhibitory effects on the bone marrow; in addition, in cases of chronic inflammation, hypoferremia, iron sequestration in macrophages and iron-restricted erythropoiesis could occur (Fry 2010; Ganz & Nemeth 2009). Inflammation could contribute to a reduction in serum iron values because of sequestration in organs such as the spleen, liver and bone marrow, contributing to functional iron deficiency and defective heme synthesis (Naigamwalla, Webb & Giger 2012). In this condition, anaemia is defined as normocytic hypochromic in the early phase, changing to microcytic hypochromic in chronic cases. The production of inflammatory mediators may increase because of the intensity of fat mobilisation, as animals in the present study lost body condition during peripartum; this period of catabolism is associated with decreased erythrogram values.

During the transition from the pregnant-non-lactating phase to the non-pregnant–lactating phase, there is an increase in the nutritional demand for milk production (Drackley & Cardoso 2014). Cows enter a state of negative energy balance and need to mobilise body fat to meet this energy deficit because of milk production (Barletta et al. 2017; Ospina et al. 2010). Seker and Unsuren (1989) noted reduced values of Ht and Hb as milk production increased in dairy cows. According to our data, milk production remained the same between 14 and 60 days postpartum, and therefore, there was no increase in milk production during the postpartum period, which is inconsistent with the hypothesis that reduction of erythrogram values occurred because of increased milk production. However, it is likely that cows with nutritional deficiencies do not increase their milk production during the early postpartum period. In addition, reduced Ht, RBC and Hb values could be related to nutritional deficiencies.

The prioritisation of nutrients during peripartum contributes to the transmigration of NEFA, proteins, immunoglobulins, glucose and calcium, amongst other nutrients, towards the mammary gland (Drackley 1999; Drackley & Cardoso 2014). There is an increase in the concentration of growth hormone and a low serum insulin concentration associated with these changes, resulting in decreased lipogenesis and a reduced ability of insulin to inhibit lipolysis, contributing to increased lipolysis (Drackley & Cardoso 2014; Fiore et al. 2017; Ji et al. 2012). Increased lipolysis results in increased mobilisation of triglycerides and NEFA to meet the high energy demand during this phase, resulting in a loss of BCS, characterising the homeorrhetic changes of the peripartum period (Drackley & Cardoso 2014; Fiore et al. 2017; Herdt 2000; Lean et al. 2013).

Our data show that the BCS at the beginning of transitional period (3.5) and at parturition (3.35) were higher than the recommend scores of 3.25 and ≤ 3.0 (Stefańska et al. 2016). The animals lost 0.45 points during the first month of lactation, which was higher than recommended by Stefanska et al. (2016), who reported that losses should not be > 0.25 during the first month to avoid impairment to the animals’ health. Cows that present a high loss of BCS during the transition period have a greater reduction in dry matter intake (Hayirli et al. 2002; Roche et al. 2009).

Studies have associated intense tissue mobilisation with immunosuppression (Contreras & Sordillo 2011; Leblanc 2012). Kalaitzakis et al. (2011) reported a decrease in Hb and Ht in cows with severe lipomobilisation compared to a group of cows without lipomobilisation, whereas Bélic et al. (2010) demonstrated a low haemoglobin concentration in obese cows after parturition because of oxidative stress and altered liver function. Rafia et al. (2012) evaluated the effect of BCS on the haematological profile of dairy cows during peripartum; cows were classified as under-conditioned (BCS: ≤ 2.75), moderate conditioned (BCS: 3–3.75) and over-conditioned (BCS: ≥ 4); and blood samples were collected 30 days prior to calving and up to 30 days postpartum. The values for Hb, Ht and RBC were lower during postpartum in the three groups compared with the values from the prepartum period; however, no differences were observed between the groups during the postpartum period. Excessive lipomobilisation causes changes in the balance between inflammatory development and resolution, favouring an excessive inflammatory response during the peripartum period (Contreras & Sordillo 2011). These haematological changes are not necessarily indicative of disease but reflect the physiological variations that occur during the transitional period (Fiore et al. 2017).

## Conclusion

Our data show that the cows in this investigation suffered reduction in selected haematological profiles during the postpartum period. Therefore, future research is necessary to elucidate the relationship between inflammatory processes and the haematological profile of dairy cows during the peripartum period to facilitate interpretation of laboratory results.
